# CHAP-child: an open source method for estimating sit-to-stand transitions and sedentary bout patterns from hip accelerometers among children

**DOI:** 10.1186/s12966-022-01349-2

**Published:** 2022-08-26

**Authors:** Jordan A. Carlson, Nicola D. Ridgers, Supun Nakandala, Rong Zablocki, Fatima Tuz-Zahra, John Bellettiere, Paul R. Hibbing, Chelsea Steel, Marta M. Jankowska, Dori E. Rosenberg, Mikael Anne Greenwood-Hickman, Jingjing Zou, Andrea Z. LaCroix, Arun Kumar, Loki Natarajan

**Affiliations:** 1 Center for Children’s Healthy Lifestyles & Nutrition, Children’s Mercy Kansas City, 610 E. 22ndSt., Kansas City, MO 64108 USA; 2 Department of Pediatrics, University of Missouri - Kansas City, Kansas City, MO USA; 3 Institute for Physical Activity and Nutrition (IPAN), School of Exercise and Nutrition Sciences, Deakin University, Geelong, Australia; 4 Alliance for Research in Exercise, Nutrition and Activity, Allied Health and Human Performance, University of South Australia, Adelaide, Australia; 5 Department of Computer Science and Engineering, University of California San Diego, La Jolla, CA USA; 6 Herbert Wertheim School of Public Health and Longevity Science, University of California San Diego, La Jolla, CA USA; 7 Population Sciences, Beckman Research Institute, City of Hope, Duarte, CA USA; 8 Kaiser Permanente Washington Health Research Institute, Seattle, WA USA

**Keywords:** ActiGraph, ActivPAL, Measurement, Physical activity, Sedentary

## Abstract

**Background:**

Hip-worn accelerometer cut-points have poor validity for assessing children’s sedentary time, which may partly explain the equivocal health associations shown in prior research. Improved processing/classification methods for these monitors would enrich the evidence base and inform the development of more effective public health guidelines. The present study aimed to develop and evaluate a novel computational method (CHAP-child) for classifying sedentary time from hip-worn accelerometer data.

**Methods:**

Participants were 278, 8–11-year-olds recruited from nine primary schools in Melbourne, Australia with differing socioeconomic status. Participants concurrently wore a thigh-worn activPAL (ground truth) and hip-worn ActiGraph (test measure) during up to 4 seasonal assessment periods, each lasting up to 8 days. activPAL data were used to train and evaluate the CHAP-child deep learning model to classify each 10-s epoch of raw ActiGraph acceleration data as sitting or non-sitting, creating comparable information from the two monitors. CHAP-child was evaluated alongside the current practice 100 counts per minute (cpm) method for hip-worn ActiGraph monitors. Performance was tested for each 10-s epoch and for participant-season level sedentary time and bout variables (e.g., mean bout duration).

**Results:**

Across participant-seasons, CHAP-child correctly classified each epoch as sitting or non-sitting relative to activPAL, with mean balanced accuracy of 87.6% (SD = 5.3%). Sit-to-stand transitions were correctly classified with mean sensitivity of 76.3% (SD = 8.3). For most participant-season level variables, CHAP-child estimates were within ± 11% (mean absolute percent error [MAPE]) of activPAL, and correlations between CHAP-child and activPAL were generally very large (> 0.80). For the current practice 100 cpm method, most MAPEs were greater than ± 30% and most correlations were small or moderate (≤ 0.60) relative to activPAL.

**Conclusions:**

There was strong support for the concurrent validity of the CHAP-child classification method, which allows researchers to derive activPAL-equivalent measures of sedentary time, sit-to-stand transitions, and sedentary bout patterns from hip-worn triaxial ActiGraph data. Applying CHAP-child to existing datasets may provide greater insights into the potential impacts and influences of sedentary time in children.

## Introduction

There is some evidence that sedentary behaviors are associated with cardiometabolic risk factors in children, such as obesity [1]. However, these associations have been more consistently observed for reported sedentary behavior and screen time than for device-measured sedentary time [1–5]. While some evidence has suggested that the accumulation of sedentary time in prolonged as opposed to sporadic, interrupted bouts, often referred to as ‘sedentary bout pattern’, may be particularly detrimental to children’s cardiometabolic health, findings have also been inconsistent [3, 6–8].

A primary methodological challenge facing sedentary pattern research revolves around the measurement of the timing and duration of sedentary bouts. Sedentary behavior is formally defined as energy expenditure ≤ 1.5 metabolic equivalents (i.e., low movement) and a seated, reclined, or lying position (i.e., posture) [9, 10]. However, numerous studies have shown the postural component is not well captured by accelerometer counts-per-minute (cpm) cut-points, leading to overestimation of breaks in sedentary time (i.e., transitioning out of a sedentary bout) and underestimation of sedentary bout durations [11–14]. Despite these limitations, hip-worn accelerometer cut-points remain the most commonly used technique for assessing children’s sedentary patterns [3]. Thus, progress in sedentary research hinges on the development of more accurate measures for capturing bouts of sedentary time from these accelerometers.

The activPAL thigh-worn accelerometer, which provides inclinometer functionality, has become the preferred tool for sedentary assessment in children because of its ability to discern sitting from standing/stepping using proprietary algorithms [15, 16]. Although the activPAL is not considered a gold standard, it is widely accepted for its ability to measure sedentary time and has been shown to have high agreement with direct observation criterion measures in a range of population and age groups, including children [15, 17, 18]. While studies are increasingly incorporating the activPAL, it is likely to take many years to generate this evidence at a large scale [19]. To accelerate this field of research, methods are needed that can improve posture classification from hip-worn accelerometers and be applied to existing and future datasets. Existing research in this area has shown that it is feasible to develop such methods, though the small number of previous studies have been limited to adults [20–22]. Thus, there is a need to develop hip-worn accelerometer posture classification methods for children to improve the quality of research investigating health impacts of children’s sedentary time and patterns [3, 6–8].

The present study aimed to develop and evaluate a novel classification method, termed CHAP-child (Convolutional Neural Network [CNN] Hip Accelerometer Posture), for measuring posture-based sedentary time in children. CHAP-child was developed to classify brief epochs (i.e., 10 s) of data from hip-worn triaxial ActiGraph accelerometers as sitting or standing, with equivalence to the activPAL (i.e., concurrent validity). The triaxial ActiGraph accelerometer was selected because it has been the most widely used device across the world for measuring children’s activity over the past decade and continues to be widely used [23]. Our evaluation of CHAP-child focused on epoch- and participant-level agreement with activPAL.

## Methods

### Participants and procedures

The present study analyzed data from the Patterns of Habitual Activity across SEasons (PHASE) study [24]. Primary schools located within 40 km of the Melbourne Central Business District, Australia, and with > 200 pupils, were stratified into tertiles of socioeconomic status (SES) using the Socio-Economic Index for Areas [25]. Schools within each SES stratum were randomly selected and invited to participate. Principals from nine schools (five high, three mid, and one low SES) agreed for their school to participate in the study. All 1270 children in Years 4 and 5 (aged 8–11 years) received an invitation to participate. Informed written parental consent for at least one component of the study was received for 326 children (25.7%). Approvals for the study were granted by the Deakin University Human Ethics Advisory Group (Health), Department of Education and Early Childhood Development, and Catholic Education Office (Melbourne) (approval identifiers: HEAG-H 13_2012 and 2020–265).

Each participant was asked to complete a physical activity assessment (simultaneous wear of the ActiGraph and activPAL) in the winter, spring, summer, and fall, up to four total measurement periods. Of the 1304 (326*4) possible ‘participant-seasons’, 586 (45%) were excluded from the present analyses due to having no valid days for which both accelerometers were simultaneously worn. The final sample comprised 718 participant-seasons from 278 participants. Participants were divided into three sets: a training dataset (*n* = 156), used to train the candidate CHAP-child models; a model selection dataset (*n* = 38), used to compare candidate models and select the final model; and a testing dataset (*n*= 84), used to evaluate the performance of the final CHAP-child model selected. This partitioning was necessary because machine learning models have been shown to overperform when applied to data from the same participants on which they were trained [26]. The model selection dataset was necessary because several models were evaluated to inform the selection of the final model. No participant was assigned to > 1 dataset. Randomization was used to achieve balance on participant age, sex, and accelerometer wear time, school SES and school identifier, and season to maximize variability in potential correlates of sedentary time within each dataset, which improves generalizability in the results [26]. Specifically, we randomly assigned participants to the training, model selection, and testing datasets, evaluated distributions of these variables, and repeating the randomization until the datasets were approximately balanced.

### Measures

#### Participant characteristics (baseline descriptive information)

Participant age and sex were assessed by questionnaire. Body mass index (BMI, kg⋅m^−2^) at the first assessment period was calculated using objective height and weight measures collected via standardized protocols, then converted to age- and sex-normed BMIz scores [27].

#### Accelerometers

Each activity assessment involved concurrent wear of a hip-mounted triaxial ActiGraph GT3X + (ActiGraph LLC, Pensacola, FL, USA) and thigh-mounted activPAL3 (PAL Technologies Ltd, Glasgow, Scotland) for up to eight consecutive days. Participants were instructed to remove the monitors during water-based activities and sleep. The ActiGraph was worn on an elastic belt and situated on the right side, along the anterior axillary line at the level of the suprailiac crest. The activPAL was enclosed in a small pocket on an adjustable elastic belt and secured at the mid-anterior position on the child’s thigh.

### Data format and pre-processing

The activPAL yielded output in an ‘events’ file that was used to label one second epochs as sitting/lying (i.e., sedentary, referred to hereon as sitting) or standing/stepping (recoded as non-sitting), which were based on activPAL’s proprietary VANE algorithm set to require ≥ 10 s in a new posture for the posture to be registered [28]. The ActiGraph yielded two types of output: (1) raw acceleration values for the three accelerometer axes were collected at 30 Hz and used at 10 Hz (10 rows/values per second), chosen since these values rarely vary over a higher frequency; (2) acceleration counts values were extracted at one minute epochs (once value each minute), which were based on ActiGraph’s counts algorithm. The raw acceleration values were used for training the CHAP-child deep learning models, and the counts values were used to determine ActiGraph non-wear time and to compare CHAP-child to current practice (i.e., the 100 cpm cut point method) in the final statistical analyses. Non-wear time was determined separately for each monitor and sleep time was determined for the activPAL, which may have been included in the data if a participant failed to remove the activPAL at bedtime. Data were only included for periods that registered as wear time for both monitors and as non-sleep for the activPAL. ActiGraph non-wear was determined by the Choi algorithm (90-min window, 30-min streamframe, and 2-min tolerance) [29, 30]. activPAL non-wear and sleep were determined by ProcessingPAL using default settings that were shown to have good validity in this population [31–33]. There were no additional wear time criteria employed for the training and model selection datasets. The final pre-processing step for creating the deep learning model inputs involved aggregating the activPAL data to 10-s epochs. An epoch was considered sedentary if ≥ 6 s were labeled sitting/lying, otherwise it was labelled as non-sitting. 10-s epochs were chosen over longer time intervals when developing CHAP-child to provide the highest possible resolution of information that may be desired in some circumstances, such as for determining the precise timing of a sit-to-stand transition. Shorter time intervals were considered but believed to be less appropriate due to (1) the previously mentioned 10-s minimum requirement in a new posture for the posture to be registered by the activPAL, a commonly used threshold used to define postural transitions [28, 34], and (2) the potential for small amounts (e.g., several seconds) of time drift between two sensors over the wear period [35].

### CHAP-child model development

Details of the machine learning architecture and training procedures have been previously published [22, 36], with additional information available at https://github.com/ADALabUCSD/DeepPostures. The python based TensorFlow platform was used. Using the training dataset (*N* = 156 participants), a deep learning CNN was developed and applied to the 10 Hz raw ActiGraph data to generate features for which values varied every 10 s. This epoch length was chosen to match the timing of the 10-s epochs in the activPAL data. The raw triaxial acceleration data provide information on monitor positioning and rotation that are used by the CNN. The CNN aimed to automatically identify features within each 10-s epoch that could differentiate between sitting (includes lying) and non-sitting.

This convolution-based approach contrasts with traditional feature engineering, which requires researchers to pre-define features in the data (e.g., mean and variance) that are expected to have predictive utility. The CNN output features were then fed into a bi-directional long short-term memory network to learn the temporal dynamics of how sitting and non-sitting epochs occur in sequence, capturing the timing of the beginning and end of periods of sitting. Lastly, a softmax layer was used to determine the probability of sitting versus non-sitting for each 10-s epoch. The predicted label for the 10-s epoch was the posture which had the higher predicted probability (i.e., probability > 0.5). Transitions can be inferred based on the beginning and end (i.e., sit to stand transition) of each sitting period/bout, but were not actually labelled by the CHAP-child model. Numerous models were trained, differing on hyperparameters (e.g., window size, number of layers and neurons). Their performance was compared in the model selection dataset, with the best-performing model being selected as the final ‘CHAP-child’ model. Selection was based on a combination of balanced accuracy for predicting A) sitting vs. non-sitting, and B) sit-to-stand transitions, with the model that maximized both values being selected.

### Post-processing and variable derivation

In the test dataset, which was used in the statistical analyses for evaluating CHAP-child, participant-level sedentary variables were scored by aggregating the (1) one second epoch activPAL labels, (2) 10-s epoch CHAP-child labels, and (3) one minute epoch ActiGraph counts data within each participant-season. The resolution of input data for the acivPAL and ActiGraph counts reflected usual practice, and the counts data were not used in shorter epochs because previous research has shown shorter epochs (e.g., 15 s) lead to an even greater overestimation of sit-to-stand transitions [11]. For these counts data, a minute was considered sedentary if the vertical axis value was > 100 cpm [37].

Sedentary bouts were defined as periods of sedentary time lasting ≥ 1 epoch, meaning that the shortest possible bout duration was 10 s for activPAL (due to the requirement of ≥ 10 s in a new posture for the posture to be registered), 10 s for CHAP-child, and one minute for the ActiGraph cpm method. A break in sedentary time, which was synonymous with a sit-to-stand transition, was always defined as any time a sedentary epoch was followed by a non-sitting epoch (no allowance for interruptions, i.e., no tolerance).

Standard participant-level sedentary time and bout pattern variables were then calculated based on the activPAL data, CHAP-child data, and 100 cpm data [37–39]. These variables included total sedentary time (minutes/day), breaks from sedentary time (number/day), time spent in sedentary bouts lasting ≥ 30 min (minutes), mean sedentary bout duration (mean of all sedentary bouts; minutes), usual bout duration (the bout duration in minutes at which 50% of sedentary time was accumulated; minutes [40]), and alpha (an individual’s distribution/slope of sedentary bout lengths based on a power law function; unitless, lower values reflect more time in prolonged bout lengths [41]).

### Statistical analyses

The statistical analyses aimed to evaluate the CHAP-child model in the testing dataset. Participant characteristics were summarized using descriptive statistics and compared between study samples (training, model selection, and testing) using two-sample t-tests for continuous variables and chi-square tests for categorical variables.

The epoch-level analyses involved the full testing dataset of 84 participants and did not employ additional wear time criteria. To assess epoch-level agreement (i.e., 10-s labels of sitting or non-sitting), CHAP-child was compared against activPAL using sensitivity, specificity, balanced accuracy (mean of sensitivity and specificity), positive predictive value (PPV), and negative predictive value (NPV). Each metric was calculated for each participant-season. Means and standard deviations (SD) were computed across participant-seasons. To assess agreement between CHAP-child and activPAL for classifying sit-to-stand transitions, sensitivity and PPV were calculated using the transition pairing method with a 1-min lag time tolerance [42]. This approach was used due to the rare occurrences of sit-to-stand transitions relative to sitting and non-sitting. The 1-min lag threshold was selected to still give credit to CHAP-child predictions that were within 1 min of the true transition as measured by activPAL, as we believed most investigations would not require accuracy timing of < 1 min. All epoch-level classification metrics were compared between sexes and across seasons.

For analyses of participant-season level sedentary pattern variables, inclusion was limited to days with ≥ 8 h of simultaneous monitor wear and participant-seasons with ≥ 3 such days. This was done to reflect data exclusion approaches commonly used in applied studies of device-measured physical activity and sedentary time, which aim to capture a reliable representation of the participant’s activity [23, 43]. Sixty-five of the 84 participants in the testing dataset met these inclusion criteria, contributing 127 participant-seasons. To assess participant-season level agreement, the sedentary variables based on CHAP-child and the 100 cpm cut-point were compared against activPAL. Performance evaluations were focused on bias (i.e., mean difference), mean absolute error (MAE), mean absolute percent error (MAPE), Spearman correlation coefficients, and concordance correlation coefficients (CCC) [44]. All correlation coefficients were interpreted as small (≤ 0.40), moderate (0.41–0.60), large (0.61–0.80), or very large (0.81–1.0) [45]. MAPEs < 25% were judged as minimally acceptable, though there are not clear guidelines for judging these values and lower values are desirable. All statistical analyses were performed in R [46].

## Results

**Fig. 1:**
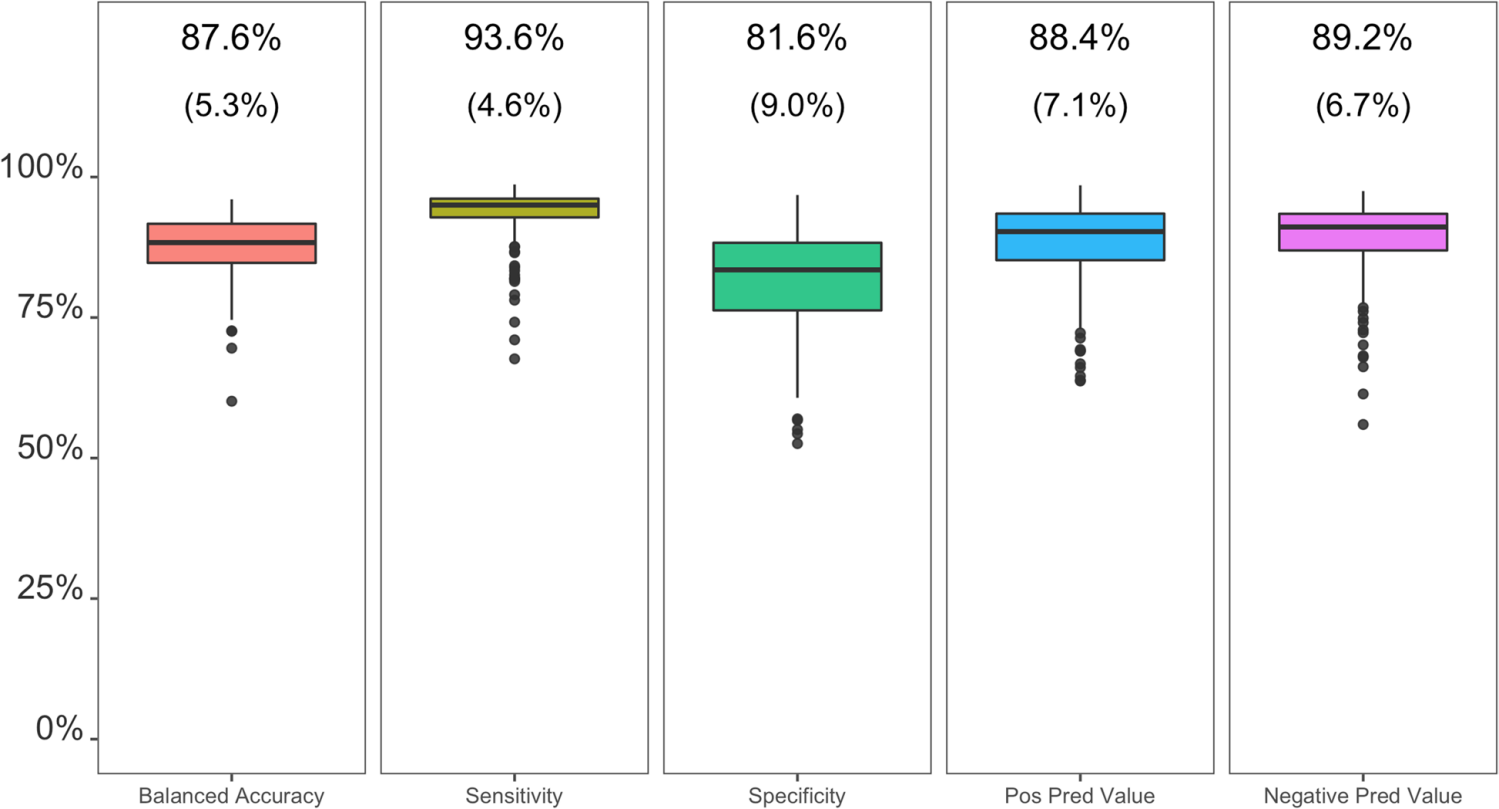
Epoch-level classification metrics for the classification of epochs as sitting (yes/no) by CHAP-child (*N* = 202 participant-seasons from 84 participants). Each metric was calculated for each participant-season and values at the top of the chart reflect the mean (SD) across participant-seasons. CHAP = Convolutional Neural Network Hip Accelerometer Posture classification method; SD = standard deviation; Balanced Accuracy = average of sensitivity and specificity; Sensitivity = correctly classified as sitting (i.e., true positives) / actual sitting; Positive Predictive Value = correctly classified as sitting (i.e., true positives) / classified sitting; Specificity = correctly classified as non-sitting (i.e., true negatives) / actual non-sitting; Negative Predictive Value = correctly classified as non-sitting (i.e., true negatives) / classified non-sitting

**Fig. 2:**
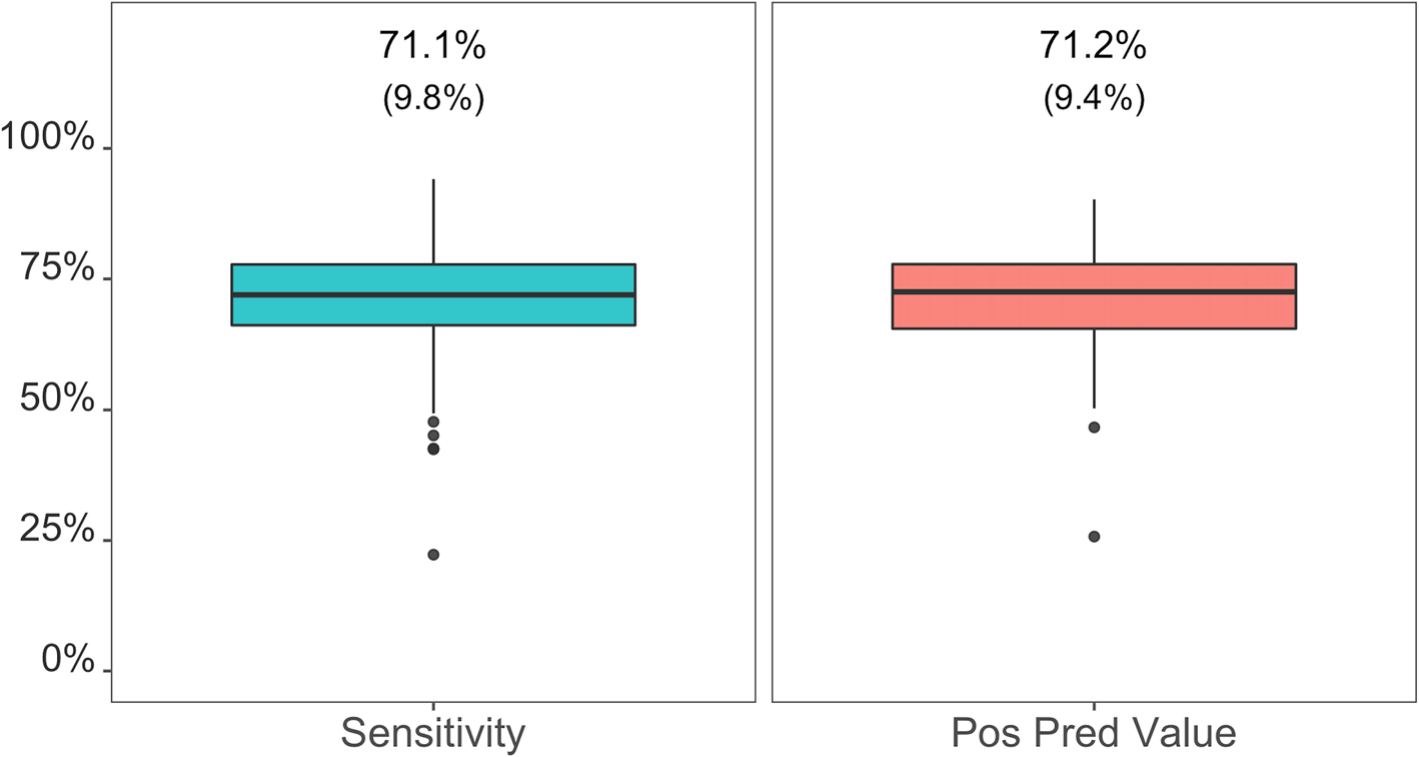
Epoch-level classification metrics for the classification of epochs as sit-to-stand transitions (yes/no) by CHAP-child evaluated using the transition pairing method with a 1-min window (*N* = 202 participant-seasons from 84 participants). Each metric was calculated for each participant-season and values at the top of the chart reflect the mean (SD) across participant-seasons. CHAP = Convolutional Neural Network Hip Accelerometer Posture classification method; SD = standard deviation; Sensitivity = correctly classified as sitting (i.e., true positives) / actual sitting; Positive Predictive Value = correctly classified as sitting (i.e., true positives) / classified sitting

The full sample comprised 278 individuals (50.7% female) with mean age of 10.5 years (SD = 0.7 years) and mean BMIz of 0.57 (SD = 1.15; Table 1). No participant characteristics differed significantly across subsamples (P ≥ 0.40). In the training and model selection datasets, each participant-season contributed a mean of 17,429 (SD = 9629) and 17,936 (SD = 9811) epochs, respectively. For the participant-season level analyses, the participant-seasons had a mean of 4.9 (SD = 1.2) valid days and 12.2 (SD = 1.6) hours per day based on time when both monitors were worn.As compared to activPAL, CHAP-child had a mean balanced accuracy of 87.6% (SD = 5.3%) across participant-seasons for classifying each 10-s epoch as sitting or non-sitting (mean Kappa = 0.76 [SD = 0.11]; Fig. 1). CHAP-child correctly classified 93.6% of actual (i.e., activPAL-labeled) sitting epochs (i.e., sensitivity) and 81.6% of actual non-sitting epochs (specificity). 88.4% of the epochs CHAP-child classified as sitting were actual sitting epochs (PPV), and 89.2% of the epochs CHAP-child classified as non-sitting were actual non-sitting epochs (NPV). CHAP-child correctly classified, within a 1-min window, 71.1% of actual sit-to-stand transitions (sensitivity), and 71.2% of the epochs CHAP-child classified as sit-to-stand transitions were actual sit-to-stand transitions (PPV) (Fig. 2). Values were slightly larger for a 5-min window (76.3% and 76.3%, respectively). No classification metrics differed by more than ± 0.8% between sexes or more than ± 5.1% across seasons.

**Table 1:** Baseline participant characteristics for each study sample (*N* = 278 participants)

	Sample			
	Training	Model selection	Testing (epoch level)	Testing (participant-season level)
Participant characteristics				
Number of participants, n	156	38	84	65
Age yr, Mean (SD)	10.5 (0.7)	10.4 (0.6)	10.5 (0.7)	10.5 (0.6)
Female, n (%)	79 (50.6%)	18 (47.4%)	44 (52.5%)	37 (56.9%)
BMIz, Mean (SD)	0.59 (1.15)	0.27 (1.09)	0.69 (1.17)	0.51 (1.16)
Low/middle socioeconomic status, n (%)	63 (40.4%)	13 (34.3%)	29 (34.5%)	21 (32.3%)
Seasons				
Number of participant-seasons, n	414	102	202	127
Fall, n (%)	90 (21.7)	19 (18.6)	44 (21.8)	24 (18.9)
Winter, n (%)	120 (29.0)	26 (25.5)	57 (28.2)	44 (34.6)
Spring, n (%)	98 (23.7)	26 (25.5)	38 (18.8)	25 (19.7)
Summer, n (%)	106 (25.6)	31 (30.4)	63 (31.2)	34 (26.8)

*BMI* Body mass index, *SD* standard deviation

Table 2 shows agreement for the participant-season level sedentary volume and pattern variables. CHAP-child estimates had MAPEs ≤ 11% for all variables except time in sedentary bouts lasting ≥ 30 min (21%), thus all MAPEs were better than minimally acceptable (< 25%). Spearman correlations and CCCs between CHAP-child and activPAL were very large (≥ 0.83) for all participant-season variables except alpha, for which *r* = 0.76 and CCC = 0.72. Conversely, 4 out of 6 MAPEs for the 100 cpm method were judged as not acceptable (31–75%) and the error was consistently in the same direction (e.g., underestimation) for all variables except breaks in sedentary time, as indicated by equivalence between the biases and MAEs. The 100 cpm method underestimated total sedentary time by a mean of ~ 20%, time in sedentary bouts lasting ≥ 30 min by ~ 75%, mean bout duration by ~ 30%, and usual bout duration by ~ 55%. The 100 cpm method overestimated alpha by ~ 52% and tended to overestimate breaks in sedentary time. Two of the six Spearman correlations were large (*r* = 0.62 for mean bout duration and *r* = 0.75 for total sedentary time) and four were moderate (*r* = 0.53–0.60), whereas two CCCs were moderate and the other four CCCs were small.

**Table 2:** Agreement of CHAP-child and the ActiGraph 100 cpm cut-point with activPAL for estimating various participant-season level sedentary pattern variables (*N* = 127 participant-seasons from 65 participants)

	Total sedentary time (min/day)	Breaks in sedentary time (num/day)	Time spent in bouts ≥ 30 min (min/day)	Mean bout duration (min)	Usual bout duration (min)	Alpha (unitless)
Descriptive statistics						
activPAL, Mean (SD)	455.1 (88.4)	82.5 (19.3)	111.5 (54.9)	5.74 (1.46)	14.68 (4.26)	1.40 (0.04)
CHAP-child, Mean (SD)	483.1 (89.0)	81.5 (17.4)	121.8 (62.3)	6.12 (1.45)	15.13 (3.92)	1.39 (0.03)
ActiGraph 100 cpm, Mean (SD)	364.3 (79.0)	91.1 (14.4)	28.1 (24.6)	4.02 (0.70)	6.53 (1.82)	2.13 (0.16)
Agreement between CHAP-child and activPAL						
Bias, Mean (SD)	28.1 (31.7)	-1.0 (8.8)	10.3 (30.1)	0.37 (0.79)	0.46 (2.17)	-0.01 (0.02)
Mean absolute error	30.6	6.8	23.4	0.61	1.48	0.02
Mean absolute percent error	6.7%	8.2%	21.0%	10.6%	10.1%	1.4%
Spearman correlation	0.93	0.87	0.85	0.84	0.84	0.76
Concordance correlation	0.89	0.88	0.86	0.83	0.85	0.72
Agreement between ActiGraph 100 cpm and activPAL						
Bias, Mean (SD)	-90.7 (58.2)	8.6 (16.1)	-83.3 (50.0)	-1.72 (1.13)	-8.15 (3.58)	0.73 (0.15)
Mean absolute error	92.2	14.9	83.3	1.75	8.15	0.73
Mean absolute percent error	20.3%	18.1%	74.7%	30.5%	55.5%	52.1%
Spearman correlation	0.75	0.57	0.55	0.62	0.60	0.53
Concordance correlation	0.48	0.49	0.14	0.24	0.10	0.01

*Bias* ActiGraph method minus activPAL, *CHAP* Convolutional Neural Network Hip Accelerometer Posture classification method, *CI* Confidence interval, *cpm* Counts per minute, *min* Minute, *num* Number, *SD* Standard deviation

## Discussion

The present findings demonstrate the concurrent validity of the CHAP-child method, based on equivalence with activPAL’s posture-based measures, for estimating total sedentary volume, sit-to-stand transitions, and sedentary bout patterns from raw triaxial acceleration data from the hip-worn ActiGraph. In contrast, the commonly used 100 cpm method showed poor validity in this study, consistent with prior research [3, 11]. Applying CHAP-child to existing datasets may refine our understanding of the potential impacts and influences of children’s sedentary patterns. It is critical to use valid measures in sedentary research that aims, e.g., to identify ideal sedentary bout patterns for supporting children’s health. Such evidence is essential for informing clear sedentary guidelines for children at the federal and global level, potentially adding to existing leisure time screen-based guidelines which have limited applicability across settings (e.g., in schools) [47, 48]. CHAP-child will also allow researchers to better assess both physical activity and sedentary patterns with one monitor, as the ActiGraph has generally been preferred over activPAL for providing valid measures of physical activity [19].

Although similar methods for estimating sedentary patterns from ActiGraph data have been developed in adults [20, 21], CHAP-child is the only such method developed specifically for use in children and appeared to perform better than these previous adult methods in their target populations. This shows the value of deep learning approaches, consistent with findings for other CHAP models that have been developed for adults and older adults [22]. All of these CHAP models have shown similar validity, though some indicators were lower for children than adults and/or older adults. For example, balanced accuracy was 87.6% for children, 92.6% for adults, and 92.9% for older adults, and sensitivity and PPV (shown in parentheses) for classifying sit-to-stand transitions were 71.1% (71.2%) for children, 74.4% (77.6%) for adults, and 83.2% (82.9%) for older adults. In spite of these minor differences, mean absolute percent errors for mean sedentary bout duration remained similar across children (10.6%), adults (12.2%), and older adults (13.0%). The slightly lower performance in children for some metrics may be related to children’s unique and sporadic movement patterns [49, 50], though a promising finding was that these differences in CHAPS performance across age groups were minimal, showing applicability to a range of populations.

As shown in the participant-season level analyses, CHAP-child performed substantially better than the ActiGraph 100 cpm cut-point. The latter generally had poor agreement with activPAL, providing further evidence of its poor validity for measuring sedentary patterns [12, 13]. Although daily number of breaks in sedentary time measured by the 100 cpm method had moderate agreement with activPAL, the slight overestimation of breaks resulted in substantial underestimation of long sedentary bouts, which led all other sedentary pattern variables to have poor validity. Thus, the CHAP-child method, but not the 100 cpm method, appears to align with a posture-based definition of sedentary bouts. This gives it greater utility for informing specific behavioral targets for interventions and guidelines (e.g., identifying maximum allowable sedentary bout durations for health).

### Applying CHAP-child

To facilitate use of CHAP-child in future studies, a Python script is freely available on the CHAP GitHub page (https://github.com/ADALabUCSD/DeepPostures). The model can be applied to raw triaxial acceleration csv files from hip-worn ActiGraphs that collected data with a sampling rate of 30 Hz (ActiGraph’s minimum setting) or more frequent (e.g., 80 Hz). The GitHub page contains README files with detailed instructions on preparing the accelerometer input files, pre-processing the data, and generating the CHAP-child predictions. Non-wear and sleep time can be accounted for during pre-processing or after running the CHAP-child predictions, with the latter post-processing approach being most efficient and recommended. The CHAP-child output is provided at the 10-s level and can be aggregated at different resolutions (e.g., at the participant level, by time of day), equivalent to what is possible with activPAL data.

### Strengths, limitations, and future directions

Study strengths included the use of a large sample of participant-seasons, evaluation of both epoch-level and participant-season level variables, and evaluation of the timing (rather than only the occurrence) of sit-to-stand transitions. The use of activPAL for the ground truth measure was advantageous for providing a higher data resolution (i.e., every second) and better accuracy than in some previous studies, such as those using person-worn cameras that capture images periodically and have difficulty capturing posture [20]. The use of deep learning builds on previous similar work that used nondeep machine learning [21], given that deep learning reflects current state of the science in machine learning. Since the study sample was limited to children aged 8–11 years, CHAP-child may not generalize to other ages of children. Future studies should test CHAP-child in a broader age range of children and adolescents, retraining the model if warranted. Although vast amounts of data exist from hip-worn triaxial ActiGraph monitors [23], there are also many large datasets for which CHAP-child is not applicable, such as those from uniaxial ActiGraph models or monitors worn on the wrist. Future studies should explore whether similar classification models can be developed for these other scenarios. The minimum sitting/upright period of 10 s applied to the activPAL could have led to failure to detect rapid (i.e., < 10 s) transitions, though it was selected to minimize false positives [34]. More work is needed to create consensus on the definition of a break in sedentary time to better inform such parameter selection. Since small amounts of time drift have been shown to occur between these monitors [35], the misalignment of epochs between monitors is a potential study limitation. However, time drift would be more likely to lead to an underestimation of agreement between methods rather than an overestimation, and the large amount of data used to train the CHAP-child model likely mitigated some of the potential negative impacts of time drift between monitors. Finally, activPAL is not a perfect measure and could have misclassified unconventional sitting postures, such as propping on the edge of a chair or kneeling on a chair, which are often observed in children [16, 51, 52]. Thus, future research evaluating CHAP-child against direct observation may be warranted.

## Conclusions

Efforts are needed to accelerate sedentary research in children, including improving understanding of potential health impacts and influences of sedentary bout patterns. The newly developed CHAP-child classification method allows researchers to derive activPAL-equivalent measures of sedentary volume and bout patterns from hip-worn triaxial ActiGraph data. This is a major advancement compared to the widely used cpm method applied to ActiGraph data, which has repeatedly been shown to provide invalid measures of sedentary patterns. CHAP-child is freely available and should be considered for use in sedentary research that employs hip-worn ActiGraph monitors in children, particularly when the focus is on siting patterns. Comparing health associations between posture- and cpm-based measures may uncover additional insights, as each reflect a different component of sedentary time (i.e., posture versus movement).

## Data Availability

The datasets used and/or analysed during the current study are available from the corresponding author on reasonable request.

## Abbreviations

BMI: Body Mass Index
CHAP: Convolutional neural network Hip Accelerometer Posture
CI: Confidence Interval
CNN: Convolutional Neural Network
CPM: Counts Per Minute
MAE: Mean Absolute Error
MAPE: Mean Absolute Percent Error
MIN: Minute
NPV: Neg Pred Value, Negative Predictive Value
NUM: Number
PHASE: Patterns of Habitual Activity across SEasons
PPV: Pos Pred Value, Positive Predictive Value
SD: Standard Deviation
SES: SocioEconomic Status
